# Research, Practice, and Policy Perspectives on Evidence-Based Programing for Older Adults

**DOI:** 10.3389/fpubh.2015.00136

**Published:** 2015-04-27

**Authors:** Marcia G. Ory, Matthew Lee Smith

**Affiliations:** ^1^Department of Health Promotion and Community Health Sciences, Texas A&M Health Science Center, School of Public Health, College Station, TX, USA; ^2^Department of Health Promotion and Behavior, College of Public Health, The University of Georgia, Athens, GA, USA

**Keywords:** older adults, evidence-based programs, chronic disease, physical activity, fall prevention, behavioral health, implementation and dissemination research

## Framing Evidence-Base Programing for Older Adults: Understanding the Interacting Influences of Research, Practice, and Policy

Demographers warn us of the “gray tsunami” approaching our global doorstep (1). Researchers are called upon to document the extent to which the growing burden of chronic conditions impacts America’s aging population and examine the uptake and effectiveness of different intervention approaches for improving the health and well-being of older adults across settings and populations (2). Working in conjunction with researchers, practitioners are asked to develop, adopt, and adapt innovative evidence-based health promotion and disease management programing that can be broadly implemented, disseminated, and sustained as appropriate in community and clinical settings (3, 4). Building on a growing research base and inventory of treatment options, policy makers are charged with identifying and supporting needed care and services that can meet the Triple Aims of health reform (i.e., better health, better health care, and better value) (5, 6).

This Research Topic on evidence-based programing for older adults reflects decades of progress by researchers, practitioners, aging service providers, and policy makers working together to understand how to help older adults achieve optimal health and well-being. Such efforts have transformed successful aging from a theoretical concept into an achievable goal (7, 8).

The scientific roots of this Research Topic are many, but our (Ory and Smith) personal interest began with the evaluation of the Administration on Aging (AoA)’s national disease prevention initiatives introduced in the 2000s, which will be described in length later in this volume (9–11). With our colleagues in the Centers for Disease Control and Prevention (CDC)-funded Healthy Aging Research Network (12, 13), we began documenting the national roll-out of evidence-based programs for older adults. We were concerned with many issues: (1) who were the major stakeholders in this national effort?; (2) what programs were being offered and who they were reaching?; (3) what could we say about the fidelity, dissemination, and sustainability of different programs?; (4) what was known about the impact of different programs in different populations and settings?; and (5) what were the best strategies for advancing the evidence-based movement?

As we explored these questions, we realized the need to look beyond single silos or perspectives to understand how researchers, program developers, and policy makers could work together more closely. Such collaborations are essential to develop, promote, and support evidence-based programing that reflects stakeholders’ perspectives and increases the likelihood of being embedded into existing structures. Ideally, evidence-based programs reflect a translation of testable research theories into key intervention elements that resonate with program adopters and intended participants. However, it is critical that interventions are seen as desirable and feasible for both organizations and intended audiences if they are to be adopted. Thus, a dynamic interaction between research and practice is desirable to ensure the appropriateness of program content and delivery, especially as they are disseminated and evaluated in different populations and settings. Similarly, it is important to examine the role the policy context plays in sustainable program success. For example, healthcare policies are theoretically designed to meet national health care goals. Researchers and practitioners can help document the benefits and consequences of current policies facilitating or impeding the growth and sustainability of evidence-based programming. Research about program effectiveness can inform new policy directions, and practitioners can provide real-world views about the practicality of different service and programing options.

**Figure 1:**
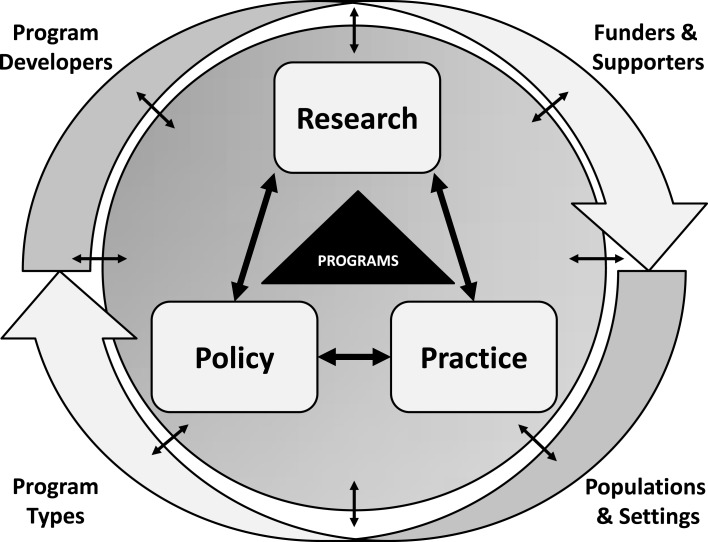
**Evidence-based programs for older adults: interacting influences and areas of study**.

In formulating this Research Topic, our collective objective was to identify the most effective programs and to understand individual, social, community, and environmental factors that influence program reach, adoption, implementation, dissemination, and sustainability. This perspective aligns with many emergent themes and frameworks in evidence-based public health and medicine such as the RE-AIM planning and evaluation framework (14, 15), the dissemination and implementation framework (16), and the movement toward translational research in promoting population health (17–20). As we framed this body of work, we created a heuristic framework (see Figure 1) to reflect the three key interacting perspectives of research, practice, and policy. Secondarily, we wanted to represent key players such as program developers and national stakeholders, the role of different program types, and the importance of specific attention to (or impacts on) different settings and populations.

## The Evidence: From Humble Beginnings

Traditional stereotypes of aging viewed older adults as inappropriate targets for community-based health promotion programs because they were believed to be uninterested in such programs and/or unable to benefit from such preventive efforts (21). However, research from the National Institute on Aging began documenting the value of a range of self-care and self-management efforts targeted at older adults (22). From a practice perspective, older adults are entitled to a variety of programs and services through the Older Americans Act (23), with Title IIID providing community-based resources for health promotion activities. In addition to providing support for congregate meals, early AoA programs focused on providing education about the importance of healthy eating and being physically active; two key risk factors for older adult health identified by national experts (24). As described in the article by the Administration for Community Living (ACL) (9), starting in the early 2000s, there was a growing impetus to develop and test best practices for health promotion/disease prevention programs. These activities coincided with the broader movement toward evidence-based practice emerging in medicine, public health, behavioral medicine (25), and complemented the recognition that education alone seldom resulted in sustained behavior change (26). Also, during this early period, there was a growing body of research about “what works” to promote healthy aging, but most studies had been conducted with limited populations and settings under controlled situations by academics and were not designed for widespread dissemination in real-world settings by practitioners (27, 28).

## Guiding the Evidence-Based Movement: Past, Present, and Future

In the past, there were few researchers involved in developing evidence-based programs for older adults, few community programs adopting these programs, few practitioners delivering these programs, and even fewer policy makers focused on strategies for guaranteeing sustainable funding streams. An initial step in promoting evidence-based programming was informing the aging services provider network about the definition of evidence-based health promotion and disease prevention and its value for practitioners and policy makers.

Toward this end, the National Council on Aging (NCOA) (29) served as the Technical Resource Center for the AoA’s new initiatives in this area. Under the leadership of Nancy Whitelaw, first Director of the Center for Healthy Aging, a variety of resources was created. These resources included the now classic briefing on “Using the Evidence-Base to Promote Healthy Aging” (30) and a series of online training modules on different aspects of evidence-based programing (31).

The articles in this Research Topics provide an excellent overview of the evolution from past to present activities, especially related to the dissemination and testing of evidence-based chronic disease self-management programs, physical activity programs, fall prevention programs, and to a lesser extent, behavioral health programs. While great strides have been made over the past three decades, there is still considerable room for improvement related to program delivery, dissemination and sustainability.

Authors of this volume were asked to reflect about future implications for research, practice, or policy. Solid groundwork has been laid, suggesting that the evidence-based movement has the foundation for even greater dissemination among an aging population. Our early work focused on the first 100,000 participants in the suite of programs referred to generically as Chronic Disease Self-Management Education (CDSME) programs. Recent statistics indicate the rapid proliferation of programs with over 300,000 persons engaged in evidence-based programs delivered through the aging services network since 2010, including more than 230,000 with CDSME alone (K. Kulinski, personal communication).

Policy changes, such as the new mandate from ACL limiting Title IIID reimbursement to evidence-based programs, will serve to increase the number of evidence-based programs disseminated to older adults through the aging services network (32). Additionally, efforts to embed evidence-based programs into existing health care systems and funding streams bode well for the long-term growth and sustainability of evidence-based programing for older adults (9, 33). As an example, the 2015 White House Conference on Aging includes policy briefs highlighting strategies for promoting health and preventing disease and injury (33).

### Perspectives from national stakeholders guiding the evidence-based program movement

While the ACL (9), in partnership with the NCOA as its technical assistance partner (29), helped mobilize the evidence-based programing movement for older adults, there are a multitude of other players at the national and regional level. The CDC has been a leader in the effort to promote public health solutions for healthy aging and fall prevention (12, 34). From a policy perspective, the Centers for Medicare and Medicaid Services are promoting policy-based research on community-based wellness and promotion programs (35). In addition to the public sphere, private foundations such as the Archstone Foundation (36), which works to prepare society for the needs of an aging population, recognize evidence-based programming as an important tool for realizing their goals. Regionally, the Health Foundation of South Florida has become a national leader in demonstrating the importance of a collaborative approach to implementing multiple evidence-based programs (37). Two interrelated themes emerge from this section: (1) the importance of involving top stakeholders in the field; and (2) the need for partnerships across research-, practice-, and policy-based agencies. Having champions well-positioned in national organizations from different aging and health sectors has helped accelerate the evidence-based movement.

### Perspectives from evidence-based program developers

This section focuses on the evolution of the evidence-based movement from the perspective of the program developers themselves Included is information regarding the processes involved in developing and taking some of the major evidence-based programs for older adults to scale, including: (1) the Stanford suite of CDSME programs (38); (2) a Matter of Balance (39); (3) stepping On (40); (4) Otago Exercise Program (41); (5) enhance fitness (42); (6) fit and strong! (43); (7) texercise (44); and (8) Program for Encouraging Active and Rewarding Lives (PEARLS) (45). Many of these programs have a long history, as exemplified by the Chronic Disease Self-Management Program (CDSMP) that has its roots as a doctoral dissertation in the 1980s (46).

The program developers generously share the lessons they learned including the importance of: (1) building programs with the end user in mind; (2) defining roles and responsibilities of partners from diverse sectors to build a culture of prevention; (3) setting up a training and certification infrastructure for widespread dissemination with fidelity; and (4) acknowledging the necessity for policy changes to provide sustainable funding streams. Additionally, the contributors express their belief in the true value of having a national data repository for real-time and continued tracking of the reach and representativeness of older participants in evidence-based programs (47). As with any intervention, a major challenge is balancing the need for program standardization (based on essential intervention elements) with adaptations desired for broader applicability to different populations and settings consistent with the latest research (48).

### Perspectives from evidence-based program networkers

The national stakeholders have helped spawn networks whose primary missions intersect with the goal of accelerating the implementation, evaluation, and dissemination of evidence-based programs for older adults. The CDC’s Healthy Aging Research Network has been instrumental in advancing science toward action and policy (12, 13). Additionally, the CDC’s National Center for Chronic Disease and Prevention has played a major role in advancing the study and application of self-management support (34), while the National Center for Injury Prevention and Control has provided a framework for identifying and intervening upon modifiable risk factors to prevent falls in later life (49). The program developers of some of the most tested and widely available evidence-based programs for older adults have recently come together to establish an Evidence-Based Leadership Council (50). Envisioning even greater numbers of participants benefiting from evidence-based programs in the future, this council is developing an infrastructure to offer technical assistance in implementation, dissemination, marketing, training efficiencies, licensing, and evaluation. In fall prevention, a national network of State Fall Prevention Coalitions has been developed to mobilize further awareness about the need for fall prevention, assist in the implementation of evidence-based programs, and help set priorities for and implement needed system change (51, 52). At the state level, state departments of public health are working collaboratively to implement a variety of evidence-based fall prevention strategies, many of which require partnerships across public health, aging, and health care sectors (53). At the local level, volunteer program facilitators and program participants are forging partnerships that help care providers and recipients (54).

It is heartening to see a variety of networks working together to promote evidence-based programming that can make a difference in the lives of older adults and their caregivers. Complemented by national stakeholders, these networks are providing the needed research and programmatic infrastructure to accelerate the evidence-based movement. They are also identifying existing policies that can facilitate or impede the broader dissemination and sustainability of evidence-based programs for older adults and addressing them accordingly.

## The Value of Research: Dissemination, Implementation, and Outcomes

In this section, we address what is being learned from national, state, and local studies about the program dissemination and implementation processes and health-related outcomes. These are best characterized as translational or pragmatic research studies conducted in real-world settings (55, 56). The major questions are often descriptive: (1) what do we know at a given point in time about who is participating in evidence-based programs?; (2) what do we know about factors associated with successful programmatic completion?; (3) what is the extent to which intended outcomes are achieved?; and (4) how do these translational efforts compare to the original randomized clinical trials or controlled studies? There is emerging research interest in understanding the spatial distribution of programs relative to need, mechanisms associated with program success, who is most likely to benefit, and the cost-effectiveness of individual and bundled programming. This research has led to the creation of guidebooks, checklists, and other tools that can help practitioners and policy makers plan strategically and evaluate different evidence-based programs.

### CDSME program dissemination through the ARRA

The American Recovery and Reinvestment Act of 2009 (ARRA) provided funds to disseminate CDSME programs in 45 states, Puerto Rico, and the District of Columbia between 2010 and 2012 (57). This initiative afforded the opportunity to address several questions about the evolution of these programs over time and their dissemination in different populations and settings. The introductory article helps set the stage by overviewing the ARRA initiative and reviewing methodological details about measure selection and data collection (47). While the database is large, containing the first 100,000 participants in the ARRA initiative, there is only limited data about participant demographics, workshop characteristics, and participant attendance. Nevertheless, we were able to address several practice- and policy-based research questions.

Even in this brief funding period, we see an evolution in the national roll-out, with participant recruitment accelerated over time (58). This was likely enabled by the establishment of an improved delivery infrastructure. Not only were subsequent cohorts of participants reached more quickly, later participants tended to be more diverse in terms of socioeconomic and health factors (58). Our explorations of the relationships between workshop characteristics and program attendance revealed the complexity of these relationships, which differed by delivery site rurality and type and also signaled the need to consider broader issues of program costs when determining ideal class sizes (59). There was confirmation in the value of 0 or orientation classes as a way of boosting class attendance (60). As expected, there was a variety of different delivery settings that enabled community practitioners to reach large numbers of participants. As expected, different delivery sites were employed in different geographic areas and attracted different types of participants, which confirmed the importance of implementing evidence-based programs through multiple channels for maximum reach and diversity (61).

This dataset also offered researchers with opportunities to examine similarities and differences in recruitment and attendance based on participant characteristics based on geographical location (i.e., rural and underserved areas) as well as racial/ethnic minority groupings (i.e., Asian, African American, and Hispanic). From these efforts, we see that participants living in rural areas are less likely to have evidence-based programs. Additionally, though individuals from rural areas represented a relatively small proportion of participants (25%), they experienced higher program completion rates (62). With this national dataset, we were also able to get a rare glimpse of Asian American participants and factors associated with their relatively high program completion rates (63). An examination of urban-dwelling African American participants showed unique patterns of delivery and attendance, which can beneficially inform future policy and practice efforts (64). A final analysis of factors associated with workshop enrollment and retention based on workshop language among Hispanic participants suggested the need for increased community capacity to deliver Spanish-led workshops (65). A common theme across all these analyses was the need for tailored interventions and strategies to attract and retain more participants from underserved areas and minority backgrounds.

### CDSME program implementation and outcomes

Adding to the emerging literature about the effectiveness of CDSMP (66, 67), several articles investigated factors influencing program implementation and outcomes associated with the suite of CDSME programs. Maintaining program fidelity is a major issue in program implementation, which can be facilitated by introducing and using streamlined fidelity checklists that provide guidance about processes before, during, and after program implementation (68). A case study approach with “successful” implementers was employed to examine organizational factors associated with long-term implementation of CDSMP in two states (69). Findings suggested the importance of utilizing strategies for addressing both internal and external factors for enhancing organizational capacity to support evidence-based programs. Specifically testing the Scheirer’s framework for sustainability (70, 71), another study examined factors necessary for sustaining CDSMP delivery with a more localized perspective. Similar sustainability factors were found, suggesting the importance of strategies such as enhancing organizational readiness, promoting program champions, providing technical assistance, and having access to participants and funding streams (72).

Several articles are focused on the adaptation of CDSMP to different settings and populations. Greater attention needs to be paid to strategies for successfully adopting CDSMP in the workplace to meet the needs of persons not typically enrolled in CDSMP programs (73). It is seen that the original CDSMP can be successfully adapted to new populations, such as cancer survivors (74). Additionally, self-management programs have been successfully delivered in other countries such as China (75), Australia, and the United Kingdom (76), although it is important to understand how the socio-political context impacts the delivery and success of such strategies.

In response to practice and policy concerns, researchers are starting to examine the cost-effectiveness of different evidence-based programs. Building on prior research documenting the cost-effectiveness of CDSMP (77), a related study examines the cost-effectiveness of CDSMP in terms of impact on quality-adjusted life years, demonstrating the added value of CDSMP (78). Knowing that practitioners and policy makers value information about program costs and cost-related outcomes, a user-friendly tool has been developed to help stakeholders customize national estimates to their local situation (79). In anticipating further cost-effectiveness studies, it is important to understand how current data might be linked to administrative health claims and challenges such linkages might present (80).

### Evidence-based fall prevention, physical activity, and mental health programs

In addition to the suite of CDSME programs, we invited articles about other evidence-based programs that address major public health issues facing the growing older adult population. With the magnitude and impact of falls on older Americans, it was especially salient to include evidence-based fall prevention programs (81–83).The CDC has been a leader in the implementation and evaluation of a comprehensive approach to fall prevention, including both community and clinical approaches. A state-wide evaluation of two community-based programs listed in the CDC compendium of evidence-based programs (84), Tai-Chi and Stepping On, demonstrates the power of such programs to improve the health and quality of life among older adults at risk for falling (85, 86). Further implementation was needed to prepare the Otago Education Program, a home-based fall prevention program, for widespread dissemination. This preparation included the development of an online training module for physical therapists (87). Broad public health dissemination of fall prevention programs requires greater appreciation of fall-related risks and the preventability of falls. An evidence-based fall prevention curriculum for community health workers has been developed to enable trusted members of the community to spread the word about fall prevention strategies and link underserved populations to evidenced-based programs (88).

In addition to programs listed in the CDC falls prevention compendium, there are evidence-based fall risk reduction programs. Analyses on A Matter of Balance, an evidence-based program originally designed to enhance confidence in preventing and managing falls (i.e., falls efficacy) and reduce fear of falling, reveal the mediating effect of increased physical activity on falls efficacy (89). A related study demonstrates significant impacts on gait speed, a major risk factor for falling and institutionalization (90). Expanding our knowledge about the general benefits of this intervention to different demographic and health subgroups, this subgroup analysis suggests the importance of targeting specific populations. It also recommends future research examining the relationship of functional performance to more distal fall outcomes (90).

Adaptations to a variety of physical activity programs for older adults are being further evaluated. Processes involved in the conversion of a practice-based lifestyle program to a formalized, testable evidence-based program are described (91). Such translations require an understanding of the benefits and challenges of both approaches as related to balancing program reach and sustainability. Studies on two adaptations of Fit and Strong! (43) have been conducted to examine: (1) program processes and outcomes involved in adapting Fit and Strong! to a lay-led model (92); and (2) the adaptation to a new population of cancer survivors (93). The translated interventions’ ability to achieve many of the previously reported outcomes shows the potency of evidence-based behavior principles to different settings and populations (94, 95). A case study of factors associated with the early adoption of enhance fitness in new settings reveals that many of the same strategies that have been used to promote sustainability of CDSMP, including assessing organizational readiness, understanding adoption across all phases from early to late, and developing new revenue streams, are also relevant to physical activity programs (96). As with CDSMP and other physical activity or fall prevention programs, the development of a fidelity tool for behavioral health programs, such as PEARLS, is important for monitoring program implementation across settings and populations (97).

## Cross-Cutting Perspectives for Evidence-Based Programing

This Research Topic identifies many cross-cutting issues essential for understanding and enhancing evidence-based program delivery, including perceptions of key stakeholders and lessons learned from the field. With a growing emphasis on translational research to address public health problems, there is now a proliferation of dissemination and implementation frameworks to guide research, practice, and policy related to program planning and implementation. As an example, the ACL has organized many of its initiatives around the RE-AIM Framework (14). An unknown issue is the actual uptake of this framework in the field. A case study of the perceived utility of the RE-AIM Framework by state agency service providers and public health partners revealed primarily positive endorsement of the framework for planning, implementation, and evaluation (98). However, there was some concern about adopting the framework as a whole, which suggests areas for further technical assistance and support.

As evidence-based programs roll-out nationally, there are questions about the ability and value of states and counties to implement multiple evidence-based programs. An early study showed that the majority of older adults lived in regions with access to only one evidence-based program (99). Since different programs attract different populations, there is benefit in having multiple programs offered in a given community and an infrastructure for cross-training to help spread programs to populations who can benefit the most.

A major theme throughout this collection of articles is the importance of engaging end users and diverse partners in the design and implementation of evidence-based programs. Thus, before implementing the STEADI Tool Kit, a clinically based fall prevention program, it was important to assess health provider’s perceptions about falls among their older patients and their current fall prevention practices (100). This information is critical for understanding the barriers and facilitators when trying to introduce the Tool Kit as a clinical resource for fall risk assessment, treatment, and referral. Further, non-traditional partners, such as the YMCA who have similar missions and delivery systems as traditional aging service providers, offer promising opportunities for collaborative efforts to disseminate evidence-based programs (101).

The importance of building strong linkages across aging, public health, and medical care sectors is becoming well-recognized and is now built into many national and state initiatives (57). Less appreciated are the roles of other sectors such as the educational system, which can help build a vibrant workforce for the implementation, evaluation, and dissemination of evidence-based programing (102).

Whereas the literature about the effectiveness of evidence-based programs for CDSME and fall prevention has blossomed in recent years with many meta-analyses (103–106), little is known about effective interventions for emotional health. A recent systematic review indicates strong evidence for skills training interventions, calling for additional evidence for other social support or physical activity intervention strategies (107).

Finally, whereas in the early years there was a lack of information about evidence-based programs, in some areas, there is now a profusion of information, making it hard for practitioners and policy makers to know where to retrieve reliable information for selecting and implementing evidence-based programs. While the national stakeholders have excellent materials on their websites, there is a new Evidence2programs Toolkit and website designed to help community-based organizations navigate through the abundance of information about evidence-based programing (108).

## Conclusion

Evidence-based programing for older adults has come of age. Past successes in identifying evidence-based programing have led to new emphases into translating research into practice and policy. There are now dedicated efforts being made to understand and incorporate best practices in building and sustaining programs over time. This includes identifying and employing strategies that will improve delivery system infrastructure for enhancing participant recruitment to, and retention in, evidence-based programs. Additionally, a national system is developing to track the spread of programs across geographical areas and monitor key factors such as delivery sites, participant characteristics, program attendance, and even limited outcome measurements.

This Research Topic identifies forces mobilizing the evidence-based movement: perspectives from program developers regarding their successes and remaining challenges; the strength of large and small networks in implementing and disseminating an evidence-based approach across aging, public health, and medical care sectors; factors influencing the dissemination, implementation, and outcomes associated with CDSME programs; the emerging literature specifying what is known about community-based falls, physical activity, and behavioral health interventions; and cross-cutting issues in the field.

This collection of articles can be seen as a reflection of the evidence-based programing of the past, present, and future. Dramatic progress has been made over the past three decades. Yet, more attention is needed to monitor and understand the dynamic interplay between specific intervention components (e.g., type, duration, and intensity) and various health, health care, and cost-related outcomes across different settings and populations. Having a better grasp on such information can guide and drive efforts to better target and tailor interventions for specific populations and settings. We recommend that future actions should be driven by a greater appreciation of interacting research, practice, and policy influences on the development, implementation, dissemination, and sustainability of evidence-based programs. It is our greatest hope that this Research Topic provides guidance to practitioners, stimulates new and unanswered research questions, and informs policy decisions that can help support and strengthen evidence-based programing for older adults.

## Conflict of Interest Statement

The authors declare that the research was conducted in the absence of any commercial or financial relationships that could be construed as a potential conflict of interest.

*This paper is included in the Research Topic, “Evidence-Based Programming for Older Adults.” This Research Topic received partial funding from multiple government and private organizations/agencies; however, the views, findings, and conclusions in these articles are those of the authors and do not necessarily represent the official position of these organizations/agencies. All papers published in the Research Topic received peer review from members of the Frontiers in Public Health (Public Health Education and Promotion section) panel of Review Editors. Because this Research Topic represents work closely associated with a nationwide evidence-based movement in the US, many of the authors and/or Review Editors may have worked together previously in some fashion. Review Editors were purposively selected based on their expertise with evaluation and/or evidence-based programming for older adults. Review Editors were independent of named authors on any given article published in this volume*.

## References

[1] National Institute on Aging and World Health Organization. Global Health and Aging. NIH Publication No. 11-7737 (2011). Available from: http://www.who.int/ageing/publications/global_health.pdf?ua=1

[2] U.S. Department of Health and Human Services. Multiple Chronic Conditions: A Strategic Frame-Work [Internet] (2014). Available from: http://www.hhs.gov/ash/initiatives/mcc/

[3] Ory MG Ahn S Towne SD Jr Smith ML Chronic disease self-management education: program success and future directions. In: Malone M Capezuti E Palmer RM, editors. Geriatrics Models of Care: Bringing ‘Best Practice’ to an Aging America. Cham: Springer International (2015).

[4] Ory MG Towne S Park CH Chodzko-Zajko W Practical strategies for implementing group exercise programs for older adults. In: Sullivan G Pomidor A, editors. Exercise in Aging Adults: A Guide for Practitioners. New York, NY: Springer International (2015).

[5] Berwick DM Nolan TW Whittington J. The triple aim: care, health, and cost. Health Aff (2008) 27:759–69. 10.1377/hlthaff.27.3.759 18474969

[6] Ory MG Ahn S Jiang L Smith ML Ritter P Whitelaw N Successes of a national study of the chronic disease self-management program: meeting the triple aim of healthcare reform. Med Care (2013) 51(11):992–8. 10.1097/MLR.0b013e3182a95dd1 24113813

[7] O’ Neill G Pruchno R Toward the 2015 white house conference on aging: creating an aging policy vision for the decade ahead. Gerontologist (2015) 55(2):179–82 10.1093/geront/gnv013 26035593

[8] Gonzales E Matz-Costa C Morrow-Howell N Increasing opportunities for the productive engagement of older adults: a response to population aging. Gerontologist (2015) 55(2):252–61 10.1093/geront/gnu176 26035601

[9] Boutaugh M Lawrence L Fostering healthy aging through evidence-based prevention programs: perspectives from the administration for community living/administration on aging. Front Public Health (2014) 2:236 10.3389/fpubh.2014.00236 PMC4410322 25964927

[10] U.S. Department of Health and Human Services. Administration for Community Living. About ACL [Internet] (2014). Available from: http://www.acl.gov/About_ACL/Index.aspx

[11] Boutaugh ML Jenkins SM Kulinski KP Lorig KL Ory MG Smith ML Closing the diversity gap: the work of the administration on aging, administration for community living. Generations (2015) 38(4): 107–18.

[12] Belza B Altpeter M Hooker SP Moni G The CDC healthy aging research network: advancing science toward action and policy for the evidence-based health promotion movement. Front Public Health (2104) 2:261 10.3389/fpubh.2014.00261 PMC4410341 25964935

[13] Wilcox S Altpeter M Anderson L Belza B Bryant L Jones DL The healthy aging research network: building capacity for public health and aging practice. Am J Health Promot (2013) 28(1):206 10.4278/ajhp.121116-CIT-564 PMC4540331 24000962

[14] Glasgow RE Vogt TM Boles SM. Evaluating the public health impact of health promotion interventions: the RE-AIM framework. Am J Public Health (1999) 89(9):1322–7. 10.2105/AJPH.89.9.1322 10474547 PMC1508772

[15] Belza B Glasgow R Toobert D RE-AIM for Program Planning: Overview and Applications. Washington, DC: National Council on Aging (2007).

[16] Damschroder LJ Aron DC Keith RE Kirsh SR Alexander JA Lowery JC. Fostering implementation of health services research findings into practice: a consolidated framework for advancing implementation science. Implement Sci (2009) 4(1):50. 10.1186/1748-5908-4-50 19664226 PMC2736161

[17] Brownson RC Colditz GA Proctor EK Dissemination and Implementation Research in Health: Translating Science to Practice. 1st ed New York, NY: Oxford University Press (2012).

[18] Brownson RC Colditz GA Proctor EK Dissemination and Implementation Research in Health: Translating Science to Practice. Oxford University Press (2012). 560 p. Available from: http://cphss.wustl.edu/Products/Documents/Bookchapter_Luke_DisseminationandImplementationResearch.pdf 10.1093/acprof:oso/9780199751877.001.0001

[19] Glasgow RE Vinson C Chambers D Khoury MJ Kaplan RM Hunter C. National institutes of health approaches to dissemination and implementation science: current and future directions. Am J Public Health (2012) 102:1274–81. 10.2105/AJPH.2012.300755 22594758 PMC3478005

[20] Institute of Medicine. The CTSA Program at NIH: Opportunities for Advancing Clinical and Translational Research. Available from: http://www.iom.edu/Reports/2013/The-CTSA-Program-at-NIH-Opportunities-for-Advancing-Clinical-and-Translational-Research.aspx 24199260

[21] Ory MG Hoffman M Hawkins M Sanner B Mockenhaupt R Challenging aging stereotypes: designing and evaluating physical activity programs. Am J Prev Med (2003) 25(3S2):164–71 10.1016/S0749-3797(03)00181-8 14552941

[22] Ory MG DeFriese GH, editors. Self Care in Later Life. New York, NY: Springer Publishing Co (1998).

[23] U.S. Department of Health and Human Services. Administration on Aging. Older Americans Act (OAA) Reauthorization [Internet] (2014). Available from: http://www.aoa.acl.gov/AoA_Programs/OAA/Reauthorization/Index.aspx

[24] Wellman NS Kamp B Kirk-Sanchez NJ Johnson PM Eat better & move more: a community-based program designed to improve diets and increase physical activity among older Americans. Am J Public Health (2007) 97(4):710–7 10.2105/AJPH.2006.090522 17329647 PMC1829349

[25] Evidence-Based Behavioral-Practice. Available from: http://www.ebbp.org/

[26] Ory MG Jordan P Bazzarre T. Behavioral change consortium: setting the stage for a new century of health behavior change research. Health Educ Res (2002) 17(5):500–11. 10.1093/her/17.5.500 12408195

[27] Ory MG Evashwick CJ Glasgow RB Sharkey JR Pushing the boundaries of evidence-based research: enhancing the application and sustainability of health promotion programs in diverse populations. In: Browning CJ Thomas SA, editors. Behavioral Change: An Evidence-Based Handbook for Social and Public Health. Edinburgh: Elsevier Churchill (2005). p. 267–93.

[28] Wilcox S Dowda M Griffin SF Rheaume C Ory MG Leviton L Results of the first year of active for life^®^: translation of two evidence-based physical activity programs for older adults in community settings. Am J Public Health (2006) 96(7):1201–9. 10.2105/AJPH.2005.074690 16735619 PMC1483857

[29] Birkel R Dessem E Eldridge S Kulinski K Lachenmayr S Spafford M Improving lives through evidence-based health promotion programs: a national priority. Front Public Health (2014) 2:255 10.3389/fpubh.2014.00255 PMC4410330 25964931

[30] Center for Healthy Aging. Using the Evidence Base to Promote Healthy Aging. Issue Brief. Number 1. Revised Spring (2006). Available from: http://www.ncoa.org/news-ncoa-publications/publications/issuebrief_1-r_usingeb.pdf

[31] Available from: http://www.ncoa.org/improve-health/center-for-healthy-aging/online-training-modules/

[32] U.S. Department of Health and Human Services. Administration on Aging. Disease Prevention and Health Promotion Services (OAA Title IIID) [Internet] (2014). Available from: http://www.aoa.acl.gov/AoA_Programs/HPW/Title_IIID/index.aspx

[33] Available from: http://www.whitehouseconferenceonaging.gov/blog/policy/post/healthy-aging-policy-brief

[34] Brady TJ Anderson L Kobau R Chronic disease self-management support: public health perspectives. Front Public Health (2014) 2:234 10.3389/fpubh.2014.00234 PMC4410343 25964925

[35] Colligan EM Tomoyasu N Howell B Community-based wellness and prevention programs: the role of medicare. Front Public Health (2014) 2:189 10.3389/fpubh.2014.00189 PMC4410406 25964913

[36] Ellen Kullman M Foundation engagement in healthy aging initiatives and evidence-based health promotion programs for older adults. Front Public Health (2014) 2:190 10.3389/fpubh.2014.00190 PMC4410412 25964914

[37] Health Foundation of South Florida. Healthy Aging Initiative (2015). Available from: http://hfsf.org/healthy_aging.aspx

[38] Lorig K Chronic disease self-management program: insights from the eye of the storm. Front Public Health (2014) 2:253 10.3389/fpubh.2014.00253 PMC4410327 25964929

[39] Haynes M League P Neault G A matter of balance: older adults taking control of falls by building confidence. Front Public Health (2014) 2:274 10.3389/fpubh.2014.00274 PMC4410326 25964938

[40] Ellen Mahoney J Stepping on: stepping over the chasm from research to practice. Front Public Health (2014) 2:148 10.3389/fpubh.2014.00148 PMC4410405 25964898

[41] Shubert TE Village or tribe? Expectations, roles, and responsibilities for effective fall prevention efforts. Front Public Health (2014) 2:163 10.3389/fpubh.2014.00163 PMC4410599 25964903

[42] Thompson M Snyder SJ Denison P Enhance fitness: a twenty year dissemination history. Front Public Health (2014) 2:270 10.3389/fpubh.2014.00270 PMC4410340 25964937

[43] Hughes S Smith-Ray RL Shah A Huber G Translating fit and strong! – lessons learned and next steps. Front Public Health (2014) 2:131 10.3389/fpubh.2014.00131 PMC4410404 25964894

[44] Riley H Texercise: the evolution of a health promotions program. Front Public Health (2014) 2:262 10.3389/fpubh.2014.00262 PMC4410332 25964936

[45] Snowden MB Steinman LE Piering P Rigor S Yip A Translating PEARLS: lessons learned from providers and participants. Front Public Health (2014) 2:256 10.3389/fpubh.2014.00256 PMC4410328 25964932

[46] Lorig K Laurin J Holman HR Arthritis self-management: a study of the effectiveness of patient education for the elderly. Gerontologist (1984) 24(5):455–7 10.1093/geront/24.5.455 6500266

[47] Kulinski K Boutaugh M Smith ML Ory MG Lorig K Setting the stage: measure selection, coordination, and data collection for a national self-management initiative. Front Public Health (2014) 2:206 10.3389/fpubh.2014.00206 PMC4410349 25964919

[48] Ory MG Resnick B Smith ML Changing behavior throughout the life-course: translating the success of aging research. Transl Behav Med (2012) 2(2):159–62 10.1007/s13142-012-0129-4 24073108 PMC3717898

[49] Kaniewski M Stevens J Parker E Lee R An introduction to the centers for disease control and prevention’s (CDC) efforts to prevent older adult falls. Front Public Health (2014) 2:119 10.3389/fpubh.2014.00119 PMC4410411 25964892

[50] Haynes M Hughes S Lorig K Simmons J Snyder SJ Steinman L Evidence-based leadership council – a national collaborative. Front Public Health (2014) 2:136 10.3389/fpubh.2014.00136 PMC4410421 25964895

[51] Beattie BL Working toward a multi-program strategy in fall prevention. Front Public Health (2014) 2:254 10.3389/fpubh.2014.00254 PMC4410622 25964930

[52] Schneider EC Beattie BL Building the older adult fall prevention movement-steps and lessons learned. Front Public Health (2014) 2:194 10.3389/fpubh.2014.00194 PMC4410413 25964916

[53] Thoreson SR Public health system perspective on implementation of evidence-based fall prevention strategies for older adults. Front Public Health (2014) 2:191 10.3389/fpubh.2014.00191 PMC4410422 25964915

[54] Rosemond CA Falling for a balance partner. Front Public Health (2014) 2:130 10.3389/fpubh.2014.00130 PMC4410408 25964893

[55] Glasgow RE Magid DJ Beck A Ritzwoller D Estabrooks PA. Practical clinical trials for translating research to practice: design and measurement recommendations. Med Care (2005) 43(6):551–7. 10.1097/01.mlr.0000163645.41407.09 15908849

[56] Tunis SR Stryer DB Clancey CM Practical clinical trials: increasing the value of clinical research for decision making in clinical and health policy. JAMA (2003) 290:1624–32 10.1001/jama.290.12.1624 14506122

[57] U.S. Administration on Aging. American Recovery and Reinvestment Act Communities Putting Prevention to Work Chronic Disease Self-Management Program Announcement (2010). Available from: http://www.cfda.gov/index?s=program&mode=form&tab=step1&id=5469a61f2c5f25cf3984fc3b94051b5f

[58] Smith ML Ory MG Ahn S Kulinski K Jiang L Lorig K National dissemination of chronic disease self-management education (CDSME) programs: an incremental examination of delivery characteristics. Front Public Health (2014) 2:227 10.3389/fpubh.2014.00227 PMC4410345 25964923

[59] Smith ML Ory MG Jiang L Lorig K Kulinski K Ahn S Workshop characteristics related to chronic disease self-management education (CDSME) program attendance. Front Public Health (2014) 2:19 10.3389/fpubh.2015.00019 25964943 PMC4410350

[60] Jiang L Smith ML Chen S Ahn S Kulinski K Lorig K The role of session zero in successful completion of chronic disease self-management program (CDSMP) workshops. Front Public Health (2014) 2:205 10.3389/fpubh.2014.00205 PMC4410344 25964918

[61] Smith ML Ory MG Ahn S Belza B Mingo CA Towne SD Jr. Reaching diverse participants utilizing a diverse delivery infrastructure: a replication study. Front Public Health (2015) 3:77 10.3389/fpubh.2015.00077 PMC4410486 25964949

[62] Towne SD Smith ML Ahn S Ory MG The reach of chronic disease self-management education programs to rural populations. Front Public Health (2014) 2:172 10.3389/fpubh.2014.00172 PMC4410417 25964906

[63] Ahn S Smith ML Cho J Jiang L Post L Ory MG Factors associated with successful completion of the chronic disease self-management program (CDSMP) among middle-aged and older Asian American participants: a national study. Front Public Health (2014) 2:257 10.3389/fpubh.2014.00257 PMC4410260 25964933

[64] Mingo CA Smith ML Ahn S Jiang L Cho J Towne SD Chronic disease self-management education (CDSME) program delivery and attendance among Urban-dwelling African Americans. Front Public Health (2014) 2:174 10.3389/fpubh.2014.00174 PMC4410424 25964907

[65] Smith ML Ahn S Jiang L Kulinski K Ory MG Factors associated with hispanic adults attending Spanish-language disease self-management program workshops and workshop completion. Front Public Health (2014) 2:155 10.3389/fpubh.2014.00155 PMC4410508 25964900

[66] Ory MG Ahn S Jiang L Lorig K Ritter P Laurent D National study of chronic disease self-management: six month outcome findings. J Aging Health (2013) 5(3):1258–74. 10.1177/0898264313502531 24029414

[67] Ory MG Ahn S Jiang L Smith ML Whitelaw N Ritter P Successes of a national study of the chronic disease self-management program: meeting the triple aim of health care reform. Med Care (2013) 51(11):992–8. 10.1097/MLR.0b013e3182a95dd1 24113813

[68] Ahn S Smith ML Altpeter M Belza B Post L Ory MG Methods for streamlining intervention fidelity checklists: an example from the chronic disease self-management program. Front Public Health (2014) 2:294 10.3389/fpubh.2014.00294 PMC4410323 25964941

[69] Paone D Factors supporting implementation among CDSMP organizations. Front Public Health (2014) 2:237 10.3389/fpubh.2014.00237 PMC4410331 25964928

[70] Scheirer MA Is sustainability possible? A review and commentary on empirical studies of program sustainability. Am J Eval (2005) 26:320 10.1177/1098214005278752

[71] Scheirer MA Dearing JW An agenda for research on the sustainability of public health programs. Am J Public Health (2011) 101(11):2059–67 10.2105/AJPH.2011.300193 21940916 PMC3222409

[72] Tomioka M Braun KL Examining sustainability factors for organizations that adopted Stanford’s chronic disease self-management program. Front Public Health (2014) 2:140 10.3389/fpubh.2014.00140 PMC4410259 25964896

[73] Smith ML Ory MG Chronic disease self-management program (CDSMP) in the workplace: opportunities for health improvement. Front Public Health (2014) 2:179 10.3389/fpubh.2014.00179 PMC4410423 25964909

[74] Risendal BC Dwyer A Lorig K Seidel RW Coombs L Ory MG Meeting the public health challenge of cancer surviorship: results of the evaluation of the chronic disease self-management program (CDSMP) for cancer survivors. Front Public Health (2014) 2:214 10.3389/fpubh.2014.00214 PMC4410485 25964922

[75] Browning CJ Yang H Zhang T Chapman A Liu S Enticott J Implementing a chronic disease self-management program into China: happy life club. Front Public Health (2014) 2:181 10.3389/fpubh.2014.00181 PMC4410613 25964910

[76] Browning CJ Thomas SA Implementing chronic disease self-management approaches in Australia and the United Kingdom. Front Public Health (2014) 2:162 10.3389/fpubh.2014.00162 PMC4410761 25964902

[77] Ahn S Basu R Smith ML Jiang L Lorig K Whitelaw N The impact of chronic disease self-management programs: healthcare savings through a community-based intervention. BMC Public Health (2013) 13:1141. 10.1186/1471-2458-13-1141 24314032 PMC3878965

[78] Basu R Ory MG Towne SD Smith ML Hochhalter A Ahn S Cost effectiveness of the chronic disease self-management program: implications for allocating community resources. Front Public Health (2014) 2:27 10.3389/fpubh.2015.00027 25964945 PMC4410335

[79] Ahn S Smith ML Altpeter M Post L Ory MG Healthcare cost savings estimator tool for chronic disease self-management program (CDSMP): a new tool for program administrators and decision makers. Front Public Health (2014) 2:42 10.3389/fpubh.2015.00042 25964946 PMC4410329

[80] Ritter PL Ory MG Smith ML Jiang L Alonis A Laurent D Linking evidence-based program participants data with medicare data: the consenting process and correlates of retrospective participant consents. Front Public Health (2014) 2:176 10.3389/fpubh.2014.00176 PMC4410409 25964908

[81] Towne SD Smith ML Yoshikawa A Ory MG Geospatial distribution of fall-related hospitalization incidence in Texas. J Safety Res (2015) 53:11–16 10.1016/j.jsr.2015.01.002 25933992

[82] Stevens JA Corso PS Finkelstein EA Miller TR. The costs of fatal and non-fatal falls among older adults. Inj Prev (2006) 12(5):290–5. 10.1136/ip.2005.011015 17018668 PMC2563445

[83] Centers for Disease Control and Prevention. Cost of Falls Among Older Adults (2014). Available from: http://www.cdc.gov/homeandrecreationalsafety/falls/fallcost.html

[84] Centers for Disease Control and Prevention. CDC Compendium of Effective Fall Prevention Interventions: What Works for Community-Dwelling Older Adults. 2nd ed (2012). Available from: http://www.cdc.gov/HomeandRecreationalSafety/Falls/compendium.html

[85] Ory MG Smith ML Parker EM Jiang L Chen S Wilson AD Fall prevention in community settings: results from implementing Tai Chi: moving for better balance in three states. Front Public Health (2014) 2:258 10.3389/fpubh.2014.00258 PMC4410325 25964934

[86] Ory MG Smith ML Jiang L Lee R Chen S Wilson AD Fall prevention in community settings: results from implementing stepping on in three states. Front Public Health (2014) 2:232 10.3389/fpubh.2014.00232 PMC4410346 25964924

[87] Shubert TE Smith ML Ory MG Clarke C Bomberger S Roberts E Translation of the Otago exercise program for adoption and implementation in the United States. Front Public Health (2014) 2:152 10.3389/fpubh.2014.00152 PMC4410425 25964899

[88] St. John JA Shubert TE Smith ML Rosemond CA Howell D Beaudoin C Developing an evidence-based fall prevention curriculum for community health workers. Front Public Health (2014) 2:209 10.3389/fpubh.2014.00209 PMC4410347 25964920

[89] Cho J Smith ML Ahn S Kim K Appiah B Ory MG Effects of an evidence-based falls risk-reduction program on physical activity and falls efficacy among oldest-old adults. Front Public Health (2014) 2:182 10.3389/fpubh.2014.00182 PMC4410414 25964911

[90] Cho J Smith ML Shubert TE Jiang L Ahn S Ory MG Gait speed among older participants enrolled in an evidence-based fall risk reduction program: a subgroup analysis. Front Public Health (2014) 2:26 10.3389/fpubh.2015.00026 25964944 PMC4410334

[91] Ory MG Smith ML Howell D The conversion of a practice-based lifestyle enhancement program into a formalized, testable program: from texercise classic to texercise select. Front Public Health (2014) 2:291 10.3389/fpubh.2014.00291 PMC4410339 25964940

[92] Lee S Ory MG Zollinger A Bhurtyal K Jiang L Smith ML Translation of fit & strong! for middle-aged and older adults: examining implementation and effectiveness of a lay-led model in central Texas. Front Public Health (2014) 2:187 10.3389/fpubh.2014.00187 PMC4410407 25964912

[93] Reynolds J Thibodeaux L Jiang L Francis K Hochhalter A Adaptation of an evidence-based physical activity intervention, fit & strong!, promotes physical activity and well being in older cancer survivors. Front Public Health (2014) 2:171 10.3389/fpubh.2014.00171 PMC4410419 25964905

[94] Ory MG Smith ML Mier N Wernicke M. The science of sustaining health behavior change: the health maintenance consortium. Am J Health Behav (2010) 34(6):647–59. 10.5993/AJHB.34.6.2 20604691 PMC3753403

[95] Belza B Prahova MP Kohn M Miyawaki CE Farren L Kline G Adoption of evidence-based health promotion programs: perspectives of early adopters of enhance fitness in YMCA-affiliated sites. Front Public Health (2014) 2:164 10.3389/fpubh.2014.00164 PMC4410415 25964904

[96] Farren L Snowden M Steinman L Monroe-DeVita M Development and evaluation of a fidelity instrument for PEARLS. Front Public Health (2014) 2:200 10.3389/fpubh.2014.00200 PMC4410416 25964917

[97] Ory MG Altpeter M Belza B Helduser J Zhang C Smith ML Perceived utility of the RE-AIM framework for health promotion/disease prevention initiatives for older adults: a case study from the U.S. evidence-based disease prevention initiative. Front Public Health (2014) 2:143 10.3389/fpubh.2014.00143 PMC4410418 25964897

[98] Towne SD Smith ML Ahn S Altpeter M Belza B Kulinski K National dissemination of multiple evidence-based disease prevention programs: reach to vulnerable older adults. Front Public Health (2014) 2:156 10.3389/fpubh.2014.00156 PMC4410420 25964901

[99] Smith ML Stevens JA Ehrenreich H Wilson A Schuster RJ O’Brien Cherry C Healthcare providers’ perceptions and self-reported fall prevention practices: findings from a large New York health system. Front Public Health (2014) 2:17 10.3389/fpubh.2015.00017 25964942 PMC4410324

[100] Ehrenreich H Pike M Hohman K Kaniewski M Longjohn M Myers G CDC and YMCA: a promising partnership for delivering fall prevention programming. Front Public Health (2014) 2:235 10.3389/fpubh.2014.00235 PMC4410333 25964926

[101] Frank JC A missing piece in the infrastructure to promote healthy aging programs: education and work force development. Front Public Health (2014) 2:287 10.3389/fpubh.2014.00287 PMC4410342 25964939

[102] Gillespie LD Robertson MC Gillespie WJ Sherrington C Gates S Clemson LM Interventions for preventing falls in older people living in the community. Cochrane Database Syst Rev (2012) 9:CD007146 10.1002/14651858.CD007146.pub3 22972103 PMC8095069

[103] American Geriatrics Society, British Geriatrics Society. AGS/BGS Clinical Practice Guideline: Prevention of Falls in Older Persons. New York, NY: American Geriatrics Society (2010).

[104] Brady TJ Murphy LM O’Colmain B Beauchesne D Daniels B Greenberg M A meta-analysis of health status, health behaviors and healthcare utilization outcomes of the chronic disease self-management program. Prev Chronic Dis (2013) 10:120112. 10.5888/pcd10.120112 23327828 PMC3547675

[105] Anderson G Chronic Care: Making the Case for Ongoing Care. Princeton, NJ: Robert Wood Johnson Foundation (2010). Available from: www.rwjf.org/content/dam/farm/reports/reports/2010/rwjf54583

[106] Snowden MB Steinman L Carlson WL Mochan KN Abraido-Lanza AF Bryant LL Effect of physical activity, social support and skills training on late-life emotional health: a systematic literature review and implications for public health research. Front Public Health (2014) 2:213 10.3389/fpubh.2014.00213 PMC4410348 25964921

[107] Stevens AB McGhee R Ory MG EvidenceToPrograms.com: a toolkit to support evidenced-based programming for seniors. Front Public Health (2014) 2:18 10.3389/fpubh.2015.00018 25984510 PMC4415232

[108] Glasgow RE Lichtenstein E Marcus AC. Why don’t we see more translation of health promotion research to practice? rethinking the efficacy-to-effectiveness transition. Am J Public Health (2003) 93(8):1261–7. 10.2105/AJPH.93.8.1261 12893608 PMC1447950

